# Astaxanthin alleviates spinal cord ischemia-reperfusion injury via activation of PI3K/Akt/GSK-*3*β pathway in rats

**DOI:** 10.1186/s13018-020-01790-8

**Published:** 2020-07-23

**Authors:** Jian Fu, Haibin Sun, Haofei Wei, Mingjie Dong, Yongzhe Zhang, Wei Xu, Yanwei Fang, Jianhui Zhao

**Affiliations:** Department of Emergency Surgery, The Second Hospital of Hebei Medical University, No.215 Heping West Road, Shijiazhuang, 050000 Hebei China

**Keywords:** Spinal cord ischemia-reperfusion injury, Astaxanthin, Oxidative stress, Inflammation, Apoptosis, Mitochondria swelling

## Abstract

**Background:**

Ischemia-reperfusion injury of the spinal cord (SCII) often leads to unalterable neurological deficits, which may be associated with apoptosis induced by oxidative stress and inflammation. Astaxanthin (AST) is a strong antioxidant and anti-inflammatory agent with multitarget neuroprotective effects. This study aimed to investigate the potential therapeutic effects of AST for SCII and the molecular mechanism.

**Methods:**

Rat models of SCII with abdominal aortic occlusion for 40 min were carried out to investigate the effects of AST on the recovery of SCII. Tarlov’s scores were used to assess the neuronal function; HE and TUNEL staining were used to observe the pathological morphology of lesions. Neuron oxidative stress and inflammation were measured using commercial detection kits. Flow cytometry was conducted to assess the mitochondrial swelling degree. Besides, Western blot assay was used to detect the expression of PI3K/Akt/GSK-3β pathway-related proteins, as well as NOX2 and NLRP3 proteins.

**Results:**

The results demonstrated that AST pretreatment promoted the hind limb motor function recovery and alleviated the pathological damage induced by SCII. Moreover, AST significantly enhanced the antioxidative stress response and attenuated mitochondrial swelling. However, AST pretreatment hardly inhibited the levels of proinflammatory cytokines after SCII. Most importantly, AST activated p-Akt and p-GSK-3β expression levels. Meanwhile, cotreatment with LY294002 (a PI3K inhibitor) was found to abolish the above protective effects observed with the AST pretreatment.

**Conclusion:**

Overall, these results suggest that AST pretreatment not only mitigates pathological tissue damage but also effectively improves neural functional recovery following SCII, primarily by alleviating oxidative stress but not inhibiting inflammation. A possible underlying molecular mechanism of AST may be mainly attributed to the activation of PI3K/Akt/GSK-3β pathway.

## Introduction

Delayed postoperative paraplegia induced by spinal cord ischemia-reperfusion injury (SCII) is still one of the critical severe and catastrophic complications of thoracoabdominal aortic surgery. The incidence of paraplegia in patients undergoing that procedure is as high as 14% [1]. A period of ischemia and blood reperfusion induces progressive damage and aggravation of nerve function [2].

Numerous studies have demonstrated that, following the primary mechanical injury to the spinal cord, oxidative stress occurs that destroys the normal balance of redox states and consequently contributes to the production of reactive oxygen species (ROS) [3, 4]. Furthermore, spinal cord mitochondria have an intrinsically higher risk of oxidative damage and overload with calcium than brain mitochondria [5]. Moreover, NADPH oxidase 2 (NOX2) is a major contributor to oxidative stress in spinal cord injury [6]. In addition, the production of large quantities of proinflammatory cytokines could also induce neuronal apoptosis and even death [7]. Growing evidence has indicated that mitochondria-derived ROS and inflammation are linked through a redox sensor known as nod-like receptor pyrin domain-containing 3 (NLRP3) [8]. Moreover, the maturation and secretion of proinflammatory cytokines require the activation of caspase-1, which together with the activation of the NLRP3 inflammasome, induces the inflammatory cascade and subsequently recruits apoptosis-associated proteins [9]. Unfortunately, despite associated studies that have been reported over 30 years, markedly effective therapy to provide neuroprotection following SCII remains lacking.

Astaxanthin (AST), a naturally occurring carotenoid pigment, is widespread in living organisms such as salmon, shrimp, microalgae, and yeast [10, 11]. AST possesses excellent biological effects, such as antioxidant activity, anti-inflammatory activity, modulation of immune responses, anti-cancer activity, and other neuroprotective properties [12, 13]. It was reported that AST treatment not only inhibited brain hemorrhagic injury across the blood-brain barrier [14] but also exhibited significant neuroprotective effects following compression spinal cord injury against neuropathic pain associated with multiple molecular targets [15, 16]. AST was also able to activate the cAMP/PKA/CREB signaling pathway in brain tissues and reduced isoflurane-induced neuroapoptosis via the phosphoinositide 3-kinases (PI3K) and their downstream target, protein kinase B (Akt) pathway, ultimately promoting axonal regeneration in the cerebral cortex and improving motor function [17], which is similar to our previous report on the neuroprotective effect of AST in mice subjected to repeated cerebral ischemia-reperfusion [18]. In addition, PI3K/Akt pathway is involved in the regulation of oxidation, inflammatory responses, and apoptosis [19]. Activation of PI3K/Akt/GSK-3β signaling pathway has been demonstrated to result in the attenuation of myocardial injury [20]. However, to our knowledge, the neuroprotective effects of AST on SCII have not been thoroughly investigated to date.

Thus, we aimed to elucidate the role of AST pretreatment in SCII injury in rats and its molecular mechanism. The current study indicated that AST pretreatment attenuates SCII injury via activation of the oxidative stress-mediated PI3K/Akt/GSK-3β pathway.

## Experiment

### Animals

Male Sprague-Dawley rats (aged 6–8 weeks, weighing 180–200 g) were obtained from the Experimental Animal Center of Hebei Medical University (animal licenses no.: SCXK [Hebei 2018-004]; animal certification no.: 190175). They were housed in a standard light-controlled room (12:12-h light/dark cycle) and maintained at a constant temperature (25 ± 1 °C) and humidity (50–60%) for at least 1 week before the experiments. Food (GB 14924.3) and water were freely available. Experiments were carried out in accordance with the guidelines for the Care and Use of Laboratory Animals published by the US National Institutes of Health and approved by the Animal Care and Use Committee of the Second Hospital of Hebei Medical University. All efforts were made to reduce the number of animals used and minimize their suffering.

### Materials

AST (purity > 98%, 25 mg/kg/day, Sigma-Aldrich, MO, USA) was resuspended in olive oil. All treatments were carried out between 08:30 and 10:30 a.m. to minimize the effects of circadian rhythm. MDA, XO, SOD, and GSH detection kits were obtained from Nanjing Jiancheng Bioengineering Institute (Nanjing, China).

### Study groups

Forty rats were randomly assigned to four groups (*n* = 10/group): (1) sham, in which animals underwent laminectomy surgery without an aortic occlusion clamp; (2) ischemia-reperfusion injury (SCII), in which rats underwent transient global spinal cord ischemia laminectomy with contusion lesion; (3) SCII+AST, in which rats received daily intragastric injections of 25 mg/kg AST for 14 days before SCII; and (4) LY+SCII+AST, in which rats received daily intragastric injections of 25 mg/kg AST and intravenous injections of LY294002 (a PI3K inhibitor, 0.3 mg/kg/day) for 14 days before SCII [21]. All cross-clamped rats underwent occlusion for 40 min before the occlusion clamp was removed, which was confirmed by the results of neurobehavioral and histopathological tests in our lab [22]. The AST dose was chosen based on previous studies [13].

#### Surgical procedure for SCII

SCII was induced in rats as previously described [22]. In brief, rats were anesthetized by intraperitoneal injection of chloral hydrate (400 mg/kg) before the surgical procedure. Core body temperature was maintained at 36 ± 0.5 °C. Before surgery, rats were placed in a supine position and were shaved from the abdomen to the leg so that the surgical area was marked and cleaned. Under aseptic conditions, a 10-cm midline incision was made, and the abdominal aorta was exposed. Before clamping, heparin was administered intravenously for 5 min for anticoagulation. The aorta was clamped approximately 1 cm below the left renal artery using two bulldog clamps. SCII was created via occlusion of the abdominal aorta for 40 min. After 40 min, the clamps were reopened, and the return of the aortic pulse was verified. Subsequently, the wound was closed in layers with silk sutures, and the rats were given an intramuscular injection of gentamicin 40,000 U to prevent infection. Finally, rats were maintained under the same pre-surgery conditions and given free access to food and water.

Rats were subjected to behavioral evaluation before surgery, 24 h, 48 h, and 72 h after surgery. After the last behavioral test was performed, 24 rats (*n* = 6/group) were anesthetized with 5% isoflurane and transcardiac perfusion with about 250 mL of cold normal saline. Next, the lumbar spinal cord (L2–5 segments) was quickly removed, and the samples were carefully dissected and then divided into two sections. One section was post-fixed using HE and TUNEL staining in order to observe cell survival and apoptosis. The other section was flash-frozen in liquid nitrogen and then stored at − 80 °C prior to further biochemical analyses, including the detection of oxidative stress and inflammatory cytokines using ELISA, as well as protein expression of NOX2, NLRP3, and PI3K/Akt/GSK-3β pathway using Western blot assay. Thereafter, the remaining 16 rats (*n* = 4/group) were decapitated, and flow cytometry was used to detect mitochondrial ROS and the degree of swelling.

#### Neurological test

The modified Tarlov scoring test was performed by two examiners blinded to the groups to evaluate the hind limb motor function of rats [22]. Briefly, the score ranged from 0 (spastic paraplegia and no movement of the lower limbs) to 5 (complete recovery and normal gait-hopping) and was defined for each rat to acquire a single value.

#### HE staining

Spinal cord tissues were fixed with 4% paraformaldehyde, washed, dehydrated, transparentized, immersed in wax, and serially cut into 5-μm-thick coronal slices. The paraffin-embedded sections were deparaffinized with xylene, graded ethanol, then mounted on slides for HE staining. The images were captured using an optical microscope and photographed using a microimaging system. The surviving intact motor neurons in the ventral horn of the spinal cord were counted and calculated as average numbers per region/rat by a histologist blinded to the groups.

#### TUNEL assay

Similarly, spinal cord tissues were embedded in paraffin as previously described [22], and 5-μm-thick sections were deparaffinized in 100% xylene and a descending ethanol series per the conventional method. The slices were stained with TUNEL mix according to the manufacturer’s instructions (Apop Tag, Oncor, MD, USA). The DNA nick was labeled, and the sections were counterstained with hematoxylin. The apoptotic index (AI) was calculated as AI = (number of positive cells/total number counted) × 100%. Cell counting was performed using high optical microscopy by a histologist blinded to the groups.

#### Biochemical examination

Spinal cord tissues were thawed and homogenized in 1:9 w/v ice-cold normal saline and centrifuged at 4000×*g* for 20 min. The upper supernatants were used for further biochemical processing.

#### Tissue malondialdehyde, xanthine oxidase, superoxide dismutase, and glutathione assays

Briefly, the malondialdehyde (MDA) level was determined by a method based on the reaction with thiobarbituric acid. Xanthine oxidase (XO) activity was determined spectrophotometrically at 293 nm based on the formation of uric acid from xanthine. Superoxide dismutase (SOD) activity was determined based on its ability to inhibit the oxidation of superoxide anions produced by a xanthine-xanthine oxidase system. One unit of SOD activity was defined as a 50% reduction of optical density at 450 nm. In addition, the GSH content of each group was measured at 412 nm. All procedures were performed under the guidance of the manufacturer’s instructions (Nanjing JianCheng Institute of Biological Engineering, China).

#### Determination of inflammatory factors

The levels of IL-1β (RLB00), TNF-α (RTA00), and IL-10 (R1000) (R&D Systems, Minneapolis, MN, USA) as well as IL-18 (EK0592, Boster Biological Technology, Pleasanton, CA, USA) were determined by ELISA kits according to the manufacturer’s instructions. They were expressed as picogram per milligram protein.

#### Determination of mitochondrial ROS production and degree of mitochondrial swelling

Sixteen rats (*n* = 4/group) were sacrificed, and their lumbar spinal cords (L2–5) were quickly excised and homogenized. Subsequently, the homogenized samples were divided into two sections. One of the sections was immediately assessed by flow cytometry at 488 nm excitation and 530 nm emission. Data were analyzed using BDFACSAria™IICell Sorter software (version 7.0, BD Biosciences). The other section was used to detect the degree of mitochondrial swelling by measuring the decrease in optical density at 520 nm, as previously described [23]. The turbidity of the reaction mixture reflected the degree of mitochondrial swelling. Freshly prepared rat spinal cord mitochondria (50 μg protein) were recorded over a period of 10 min at 25 °C in 200 μL of medium containing 250 mM sucrose, 5 mM KH_2_PO_4_ and 3 mM sodium succinate (pH 7.2).

#### Western blot assay

Briefly, the frozen spinal cord samples were processed, and the proteins were extracted (Applygen Technologies, Beijing, China) based on our previously reported protocol [20]. After determining the protein concentration, equal amounts of protein per group were resolved by 10% sodium dodecyl sulfate-polyacrylamide gel electrophoresis and transferred to a PVDF membrane (Millipore, Billerica, MA, USA), which was blocked by 5% skim milk for 1 h at room temperature. The PVDF membranes were subsequently incubated with primary antibodies (all were purchased from Abcam) overnight at 4 °C: anti-NOX2 (1:500), anti-NLRP3 (1:1000), p-Akt (1:1000), Akt (1:1000), p-GSK-3β (1:1000), GSK-3β (1:1000), and β-actin (1:1000). On the next day, PVDF membranes were washed in phosphate-buffered saline with 0.1% Tween-20 (PBST) and then incubated with a peroxidase-conjugated goat anti-rabbit IgG secondary antibody (1:1500, Millipore) for 2 h at room temperature. Detection of proteins was performed, and the levels were analyzed via imaging software (Bio-Rad Co. Ltd., Hercules, CA).

#### Statistical analysis

All data per group are expressed as the mean ± standard deviation (SD) and were analyzed using GraphPad Prism Progress, version 6.0 (GraphPad Software, Inc., La Jolla, USA). The significance of differences between the groups was determined by one-way ANOVA followed by Tukey’s HSD post hoc *t* test for multiple comparisons. *P* < 0.05 was considered as the significant difference.

## Results

### AST improved SCII-induced hind limb motor function deficit

**Fig. 1:**
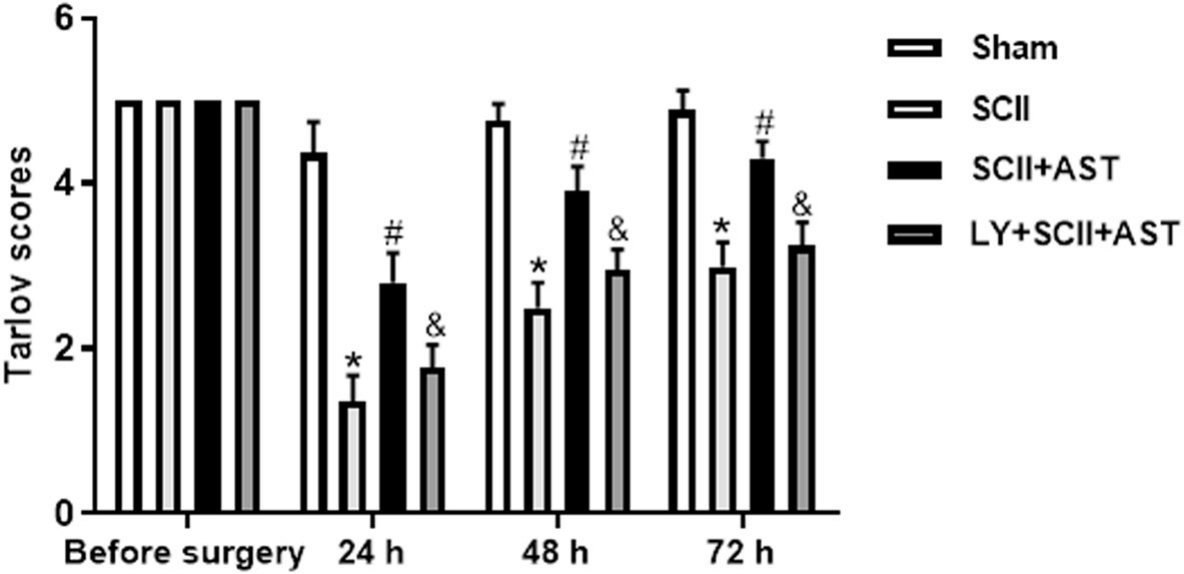
AST promoted hindlimbs motor functional recovery after SCII. Neurological functional assessment was performed from before surgery to 72 h after SCII by the Tarlov scoring system. Values are mean ± SD (*n* = 10/group). ^*^P < 0.05 versus sham group, ^#^*P* < 0.05 versus SCII group, ^&^*P* < 0.05 versus SCII+AST group

To evaluate the effect of AST on functional recovery in SCII rats, hind limb motor function was recorded using the Tarlov scoring system (Fig. 1). All rats exhibited normal movement and scores before laminectomy surgeries. In the SCII group, a significant similar decline in the Tarlov score (average of 1–2) and severe paralysis were recorded at 24 h after injury, which showed the accuracy of modeling SCII. Next, behavioral assessments of the injured rats showed a gradual increase in the Tarlov score, indicating the occurrence of a spontaneous partial recovery of function. Interestingly, the hind limb function tests of the rats in the SCII+AST group gave higher scores than those of the SCII group after 72 h. However, this protective effect of AST was found to be reversed by cotreatment with LY294002.

### AST reduced SCII-induced neuron apoptosis

**Fig. 2:**
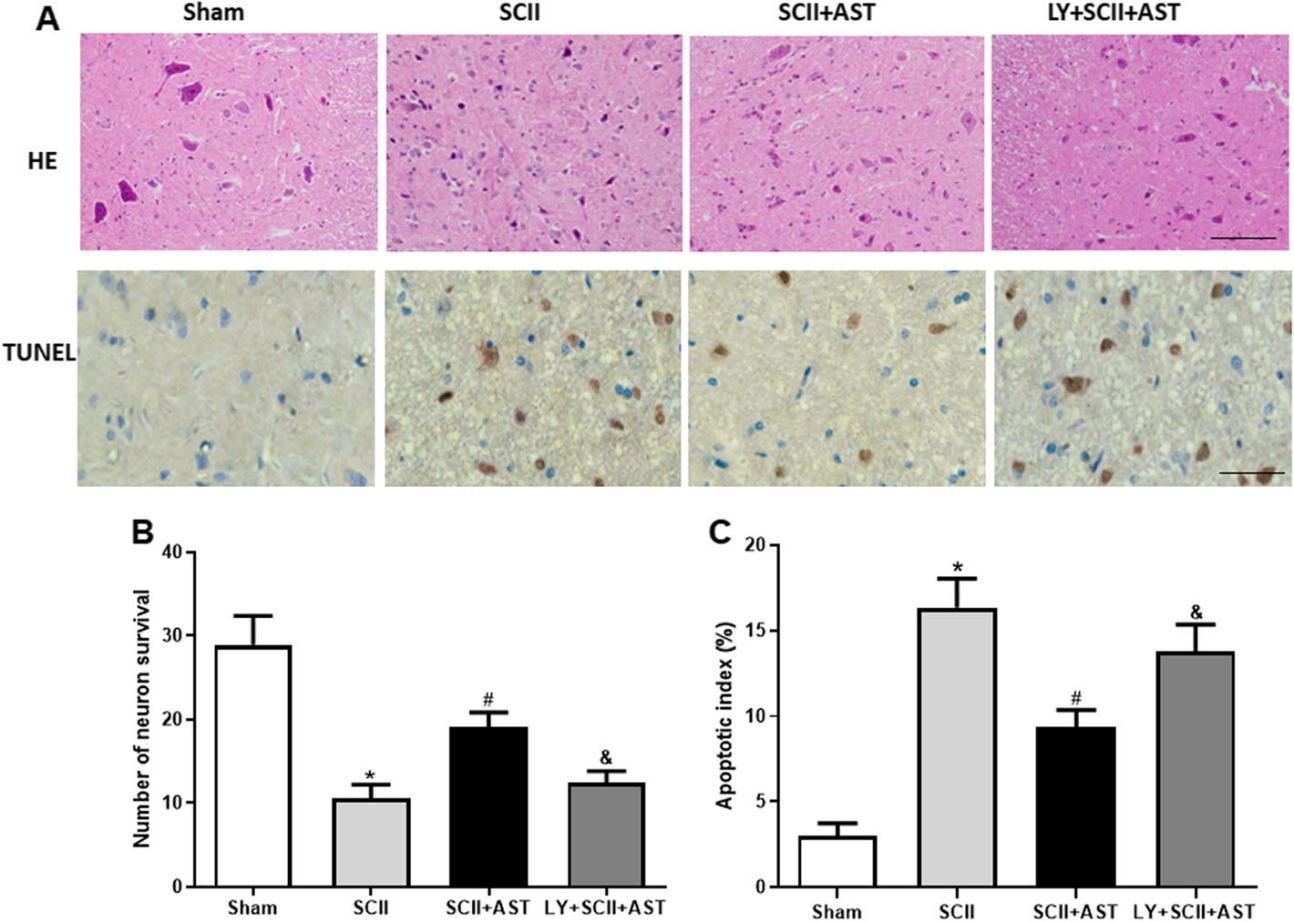
AST preserved neuron survival and inhibited cell apoptosis in the spinal cord after SCII. **a** HE and TUNEL staining were performed in different groups. The number of neuron survival and the apoptotic index of anterior horn neurons are shown in **b** and **c**, respectively. Scale bar = 200 μm. Values are mean ± SD. ^*^P < 0.05 versus sham group, ^#^*P* < 0.05 versus SCII group, ^&^*P* < 0.05 versus SCII+AST group

To observe the neuroprotective effect of AST on histopathology of the spinal cord in SCII rats, HE and TUNEL staining were performed. Notably, cells with Nissl-positive neuron were abundantly detected, and a centrally located nucleus was observed in the ventral horn of the spinal cord 72 h after injury in the sham group. However, compared with the sham group, the numbers of Nissl-positive neurons from the SCII group were lower and they displayed morphological deformation, including shrunken cell bodies and pyknotic nuclei (Fig. 2a). As expected, the number of surviving neurons was higher in the SCII+AST group compared to the SCII group (Fig. 2b). Compared with the SCII+AST group, pretreatment with LY294002 was found to counteract the effect of AST on the spinal cord.

Similarly, there were almost no TUNEL-positive cells in the sham group, while several brown-stained positive cells were observed in the SCII and the LY+SCII+AST groups. Moreover, AST pretreatment reduced the number of apoptotic cells in SCII rats (Fig. 2c).

### AST inhibited SCII-induced oxidative stress

**Fig. 3:**
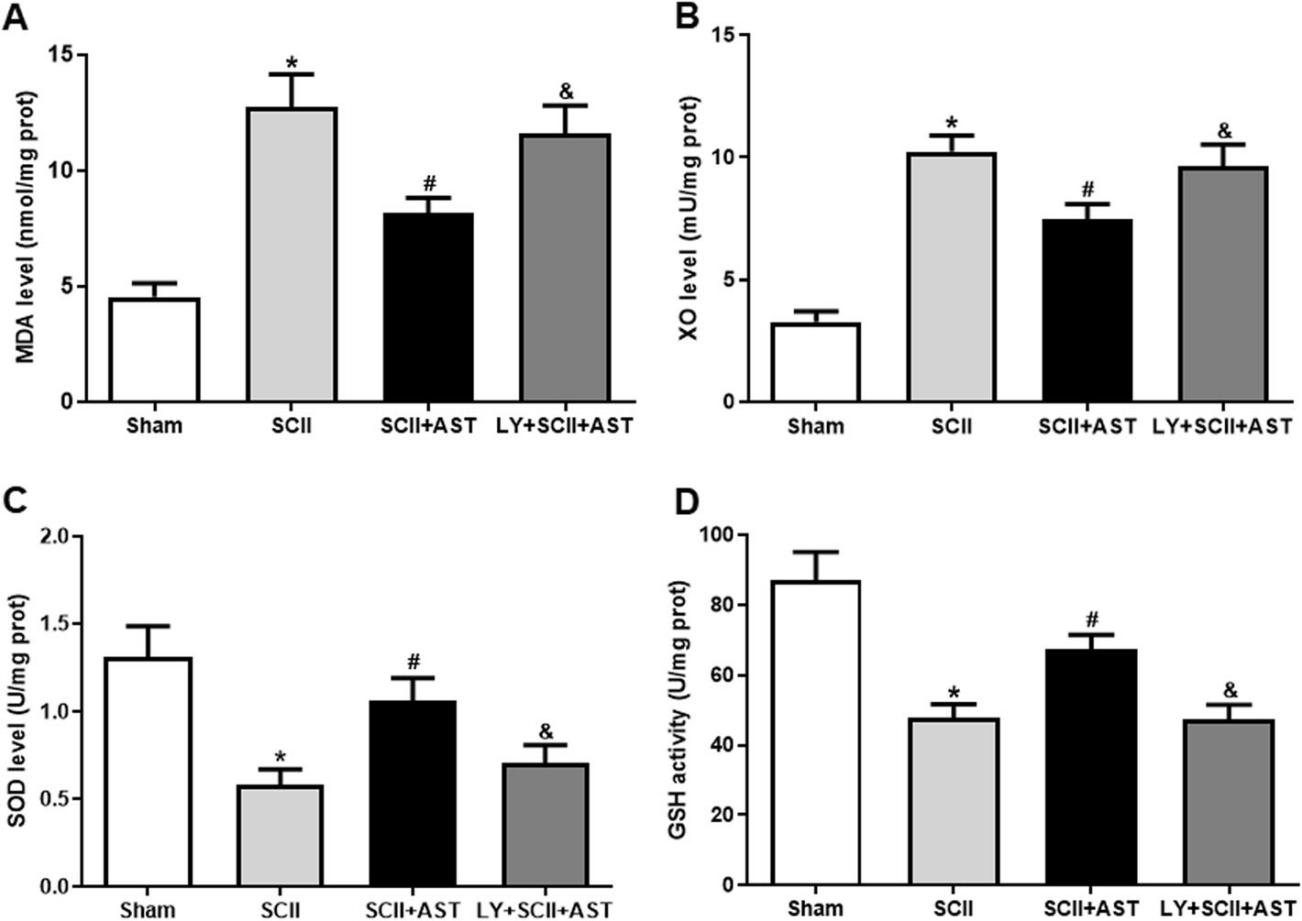
AST mitigated oxidative stress at 72 h after SCII. MDA (**a**) and XO (**b**) levels were increased, while SOD (**c**) and GSH (**d**) activities were decreased in the SCII group. In contrast, the AST pretreatment group effectively restored these changes. Values are mean ± SD (*n* = 6/group). ^*^P < 0.05 versus sham group, ^#^*P* < 0.05 versus SCII group, ^&^*P* < 0.05 versus SCII+AST group

To evaluate how AST exerts neuroprotective effects, the oxidative (MDA, XO) levels and antioxidative (SOD, GSH) activities in spinal cord homogenates were detected simultaneously. The levels of MDA and XO were higher in the SCII group. However, compared with the SCII group, the levels of MDA and XO in the SCII+AST group were remarkably lower, which means that AST strikingly mitigated the oxidative stress resulting from SCII-induced injury (Fig. 3a, b).

Concurrently, the activities of the antioxidative cytokines SOD and GSH were observed in all groups. As expected, compared with the sham group, there was a significant decrease in the levels of SOD and GSH in the SCII group. Interestingly, compared with the SCII group, the activities of SOD and GSH were higher in the SCII+AST group. However, this protective effect of AST was found to be counteracted by LY294002 cotreatment (Fig. 3c, d).

### AST partially inhibited SCII-induced inflammatory responses

**Fig. 4:**
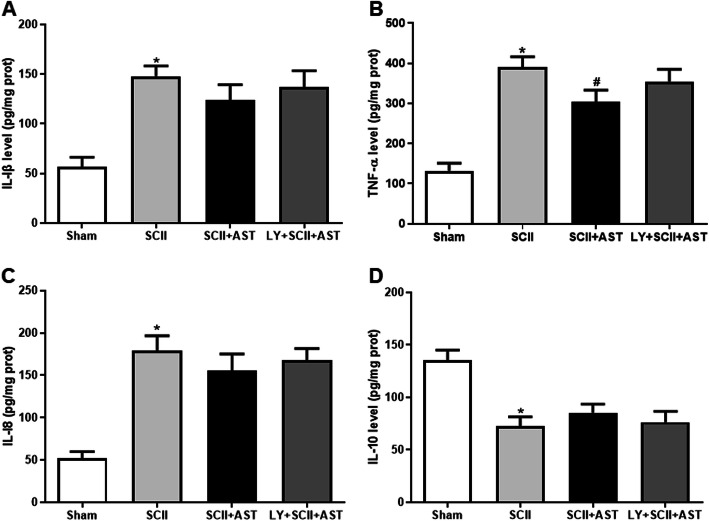
AST partially inhibited proinflammatory cytokines at 72 h after SCII. The levels of IL-1β (**a**), TNF-α (**b**), and IL-18 (**c**) were substantially increased, and IL-10 (**d**) was decreased in the SCII group compared with the sham group. Although pretreatment with AST could moderately reverse the above changes in cytokines, only the level of TNF-α was significantly decreased. Values are mean ± SD (*n* = 6/group). ^*^P < 0.05 versus sham group, ^#^*P* < 0.05 versus SCII group

Compared with the sham group, the levels of proinflammatory cytokines (IL-1β, TNF-α, and IL-18) were higher and the level of the anti-inflammatory cytokine (IL-10) was lower in the SCII group (Fig. 4). Nevertheless, compared with the SCII group, although pretreatment with AST could moderately reverse the above changes in cytokines, only the level of TNF-α was significantly decreased (Fig. 4b). It is worth noting that all of inflammation factors abovementioned were not significantly reversed by cotreatment with LY294002.

### AST suppressed SCII-induced mitochondrial ROS production and the degree of mitochondrial swelling

**Fig. 5:**
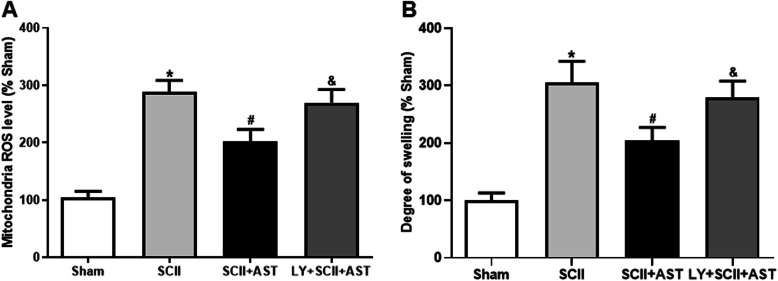
AST preserved the mitochondria further injured in the spinal cord 72 h after SCII. **a** Mitochondrial ROS production and **b** degree of mitochondrial swelling were markedly increased in the SCII group, and AST effectively suppressed these changes. Values are mean ± SD (*n* = 4/group). ^*^P < 0.05 versus sham group, ^#^*P* < 0.05 versus SCII group, ^&^*P* < 0.05 versus SCII+AST group

Compared to the sham group, mitochondrial ROS generation was significantly higher in the SCII group, whereas the mitochondrial ROS production was notably decreased in the SCII+AST group (Fig. 5a). Meanwhile, the degree of mitochondrial swelling in the isolated spinal cord mitochondria was detected by measuring the change in absorbance of the suspension at 520 nm (Fig. 5b). The degree of mitochondrial swelling was more severe in the SCII group than that in the sham group. Notably, AST pretreatment substantially inhibited mitochondrial swelling as compared with the SCII group. On the contrary, this protective effect of AST was found to be counteracted by LY294002 cotreatment.

### AST inhibited NOX2 level but not NLRP3 level of SCII rats

**Fig. 6:**
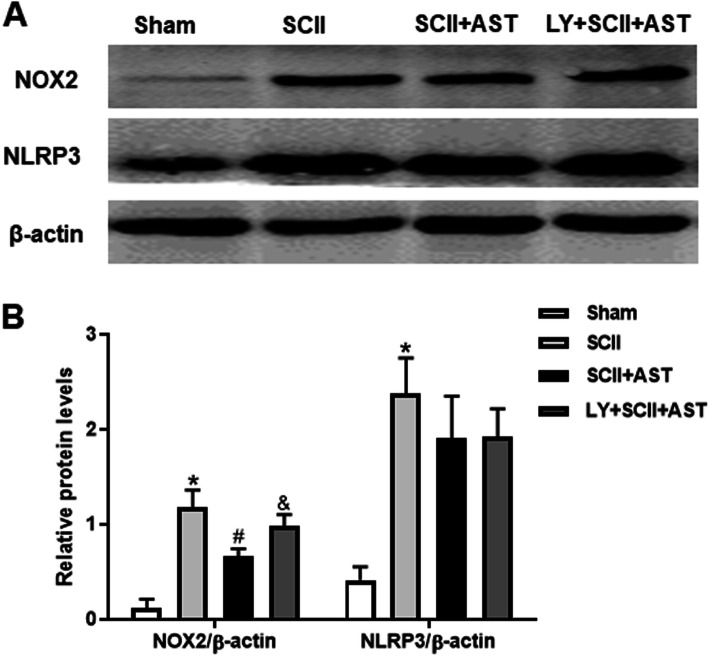
AST effectively inhibited NOX2 levels but not NLRP3 levels in the spinal cord 72 h after SCII. **a** The quantification graphs of Nrf2 and NLRP3 proteins in the spinal cord were showed using Western blot analysis. **b** All values were analyzed. Values are mean ± SD (*n* = 6/group). ^*^P < 0.05 versus sham group, ^#^*P* < 0.05 versus SCII group, ^&^*P* < 0.05 versus SCII+AST group

To further investigate the effects of AST on the molecular expressions of the oxidative stress cytokine (NOX2) and inflammatory index (NLRP3), Western blot analysis was performed. Strikingly, there were elevations of NOX2 protein and NLRP3 protein expressions in the SCII group when compared with the sham group (Fig. 6). Interestingly, the NOX2 level was markedly reduced in the SCII+AST group compared to the SCII group, while there was no significant difference in the NLRP3 levels between the SCII group and SCII+AST group, although there was a decline in the SCII+AST group. Accordingly, the level of NOX2 was reversed by cotreatment with LY294002, whereas there was no difference in the NLRP3 level.

### AST activated the PI3K/Akt/GSK-3β signaling pathway

**Fig. 7:**
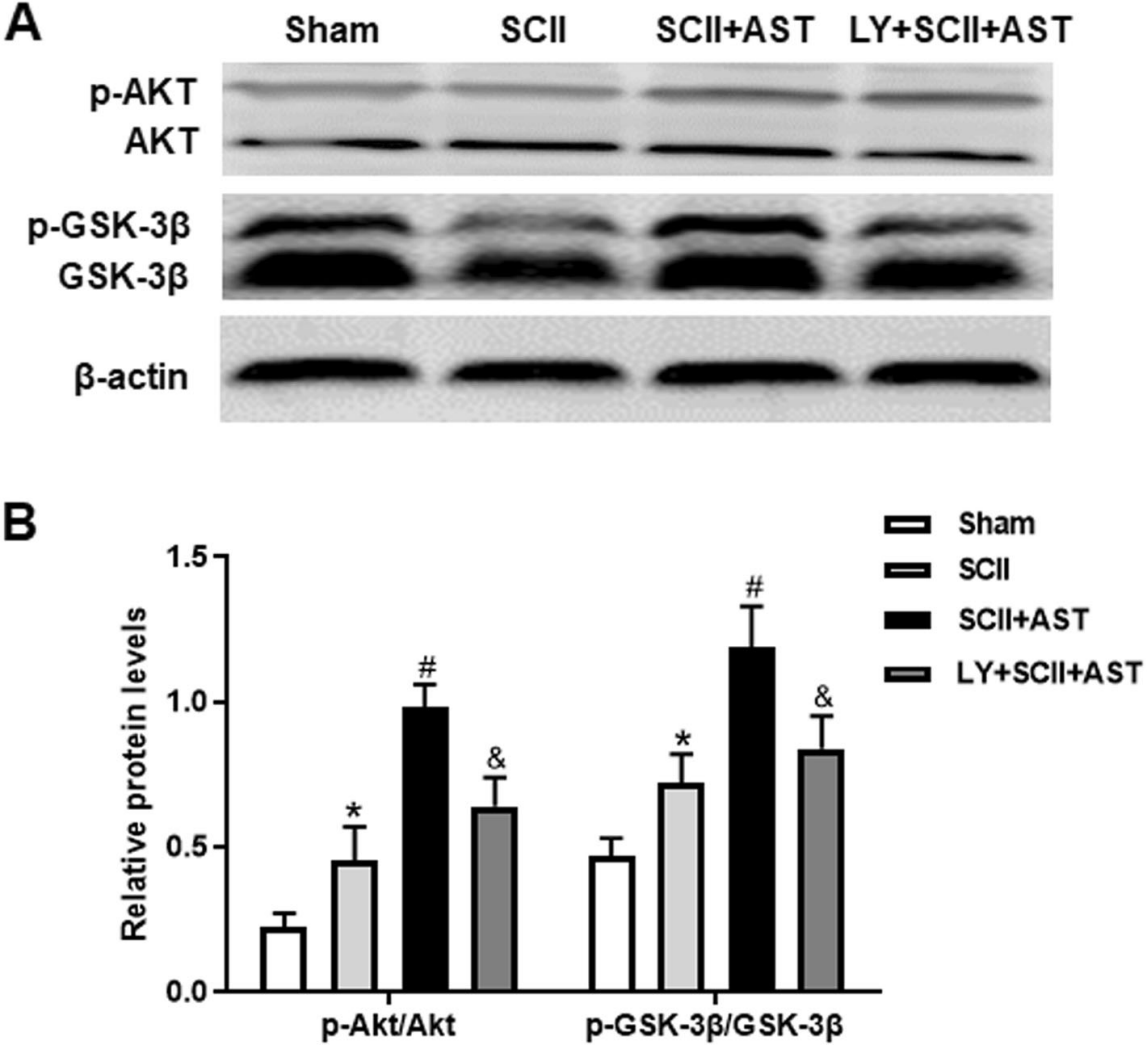
AST activated the PI3K/Akt/GSK-3β signaling pathway. **a** The quantification graphs of p-Akt, Akt, p-GSK-3β, and GSK-3β proteins in the spinal cord were showed using Western blot assay. **b** All values were analyzed. Values are mean ± SD (*n* = 6/group). ^*^P < 0.05 versus sham group, ^#^*P* < 0.05 versus SCII group, ^&^*P* < 0.05 versus SCII+AST group

To investigate the underlying mechanism by which AST protects rats against SCII, we first detected the p-Akt and Akt levels using Western blotting assay; the change in the p-Akt/Akt ratio was slightly increased in the SCII group and was further increased in the SCII+AST group. Meanwhile, this upregulation was notably reversed by LY294002 cotreatment (Fig. 7). Next, the change in the p-GSK-3β/GSK-3β ratio showed nearly the same trend, which indicates that AST may exert its protective effects on SCII rats via activating the PI3K/Akt/GSK-3β signaling pathway.

## Discussion

In the present study, we observed that AST pretreatment effectively improved motor functions and ameliorated pathological damages by reversing histological changes in the spinal cord. Furthermore, AST significantly inhibited oxidative stress and attenuated mitochondria swelling degree following the SCII. More importantly, PI3K/Akt/GSK-3β signaling pathway was found to be associated with the protective effects of AST on SCII-treated rats.

The segmental blood supply of the spinal cord and relatively poor collateral circulation facilitate ischemia damage. SCII may further aggravate spinal cord damage. Various studies introduced a pathophysiological mechanism, associated with increases in oxidative stress and inflammatory processes with apoptosis, which plays a pivotal role in SCII [2, 22, 24]. Meanwhile, oxidative stress and mitochondrial dysfunction not only result from spinal cord injury, but may directly contribute to SCII [25]. Furthermore, complex pathological mechanism changes during reperfusion injury are correlated with time and compliance [2], suggesting the importance of the involvement of early intervention in secondary damage after spinal cord injury to impede the aggressive apoptosis process.

The cell membranes of spinal cord neurons are readily susceptible to oxygen free radical attacks. After SCII, the lipid peroxidation reaction was dramatically activated, while antioxidative activities strikingly decreased [26]. Therefore, decreasing MDA levels and increasing SOD activity have been shown to significantly attenuate SCII. In addition, an obvious inflammatory response develops within several hours after injury and is characterized by the activation of proinflammatory cytokines and infiltration of neutrophils [27]. Numerous studies have reported that typical proinflammatory cytokines, such as IL-1β, TNF-α, and IL-18, are major mediators of spinal cord injury or in the pathological mechanisms of secondary damage [28]. Moreover, IL-10 also mediates the anti-inflammatory response in the spinal cord [29]. Hence, the aforementioned factors may easily contribute to spinal cord injury during the reperfusion period. However, thus far, neuroprotective agents with respect to SCII have not been systematically investigated.

AST, a dietary supplement with no significant adverse effects, is being investigated individually across a broad range of clinical applications, including cardiovascular health, acute pancreatitis, and neuropathic pain, all of which are associated with oxidative stress and inflammation in their pathogenesis [10, 16]. Besides, AST has a therapeutic role in preserving cognitive function by promoting or maintaining neural plasticity in aged patients and in neurodegenerative disease [30]. It has also been shown to maintain mitochondrial integrity and function and ameliorate oxidative stress in skeletal muscle injury [31]. Recent studies that reported AST shows prominent antioxidant and/or anti-inflammatory properties in different models of ischemia and reperfusion injury confirmed its protective action, such as in steatotic liver, muscle, and brain tissue [18, 32–34]. Based on the above characteristics of AST, it seemed to be a desirable candidate for further exploration to elucidate its therapeutic potential in SCII.

Given the numerous lines of evidence have implicated PI3K/Akt signaling pathway in modifying oxidative stress, GSK3β is a key active enzyme associated with downstream of Akt. Phosphorylation of GSK3β via Akt maintains this enzyme an inactive state and protects against tissue ischemic injury [35]. Thus, we hypothesize that PI3K/Akt signaling may act as an important antioxidant mechanism for regulating AST-induced neuroprotection following SCII.

As expected, we observed there were worse hind limb motor scores and that fewer neurons survived at 72 h after reperfusion in SCII rats compared to the sham group. Furthermore, oxidative products (MDA, XO, and mitochondrial ROS) and proinflammatory cytokines (IL-1β, TNF-α, and IL-18) were dramatically elevated in SCII rats, which is similar to our previous reports [22]. Interestingly, AST pretreatment substantially attenuated SCII-induced neurological dysfunction at various time points after reperfusion and mitigated neuronal apoptosis in the spinal cord while this effect was partially reversed when AST and LY294002 (a PI3K inhibitor) combined, which indicates that ROS-induced apoptosis after SCII is probably regulated by PI3K/Akt signaling pathway. In addition, the beneficial effects of AST pretreatment against SCII were associated with decreased levels of oxidative products (MDA, XO, and mitochondrial ROS) and proinflammatory cytokines (TNF-α), as well as increased activities of endogenous antioxidant enzymes (SOD and GSH). As to the other inflammatory cytokines, although a reverse tendency was demonstrated in the AST-treated group, such as a decrease in the IL-1β and IL-18 levels and an increase in the anti-inflammatory cytokine IL-10 level, there were no significant differences when compared with the SCII group.

Moreover, we also evaluated NOX2 and NLRP3 protein expressions, which are important factors that prompt the expressions of many oxidant stress and inflammation [36, 37]. Although we found that SCII indeed caused NOX2 and NLRP3 activation in SCII rats, AST pretreatment did not simultaneously inhibit NOX2 and NLRP3 inflammasome activation, which is consistent with our results for the above proinflammatory cytokines. Therefore, the current results further confirmed our speculation that AST suppresses SCII-stimulated ROS production, perhaps mainly because the inhibition of NADPH oxidase activity enhances antioxidative stress capabilities rather than inhibiting the NLRP3-related inflammatory pathway. The aforementioned results suggest that AST might produce a protective effect against SCII in rats, primarily via antioxidative stress activity rather than the simultaneous anti-inflammatory response.

In order to further explore the possible molecular mechanism underlying the improved motor function of SCII rats pretreated with AST, we administered the PI3K inhibitor LY294002 as a comparative study and detected the levels of PI3K/Akt pathway-related proteins, including the ratios of p-Akt/Akt and p-GSK-3β***/***GSK-3β. Similarly, these therapeutic effects were partially abolished when AST was combined with LY294002. This result might be explained that the nerve tissue had the capability of synthetizing and secreting some antioxidant enzymes to resist SCII-induced oxidative damage, although this capability did not satisfied the demand of cellular antioxidant defense [38]. After administration of AST to the SCII rats, the PI3K/Akt/GSK-3β signaling pathway associated with antioxidant stress was activated, which further enhanced antioxidative capability and then promoted nerve regeneration. Taken together, AST-induced nerve functional recovery may be mediated via the activation of PI3K/Akt/GSK-3β pathway.

## Conclusion

AST ameliorated SCII-induced oxidative stress and mitochondrial injury, as well as neuronal apoptosis in rats. Importantly, activation of PI3K/Akt/GSK-3β pathway-mediated antioxidative stress could play a critical role in the protective effects associated with AST pretreatment. Collectively, the data demonstrate that AST has the potential to serve as a therapeutic candidate for patients with SCII.

## Data Availability

All datasets on which the conclusions of this report rely are available on reasonable request.

## Abbreviations

SCII: Ischemia-reperfusion injury of the spinal cord
AST: Astaxanthin
ROS: Reactive oxygen species
NOX2: NADPH oxidase 2
NLRP3: Nod-like receptor pyrin domain-containing 3
PI3K: Phosphoinositide 3-kinases
Akt: Protein kinase B
SD: standard deviation
